# Electron microscopy analysis of femtosecond laser-assisted capsulotomy before and after lens fragmentation

**DOI:** 10.1038/s41598-021-04054-5

**Published:** 2021-12-24

**Authors:** Wolfgang J. Mayer, Andreas Ohlmann, Anna Schuh, Siegfried Priglinger, Thomas Kohnen, Mehdi Shajari

**Affiliations:** 1 Department of Ophthalmology, University Eye Hospital, Ludwig-Maximilians-University, Mathildenstr. 8, 80336 Munich, Germany; 2 Department of Ophthalmology, Goethe-University, Frankfurt am Main, Germany

**Keywords:** Eye diseases, Lens diseases

## Abstract

Studying anterior lens capsule cutting edge profiles from femtosecond laser-assisted capsulotomy procedures performed before and after lens fragmentation. Twenty eyes (10 patients) with age-related cataract underwent femtosecond laser-assisted surgery (FLACS) using the Ziemer Z8 platform. First step of laser surgery was either capsulotomy (group first) or fragmentation (group second). One eye of each patient was assigned randomly, the second eye treated with the different sequence of procedures. After anterior capsule removal, tissue was fixed in cacodylate-buffered solution and cutting-edge profiles were analysed using scanning electron microscopy (SEM). All cases had cataract grade 2 and 3 based on LOCS III grading. SEM analysis showed more smooth edges in the first group, especially in cases with pseudoexfoliation (*P* = 0.037); more tags and bridges and a significant number of staggered cutting patterns (7 out of 10 cases) in the second group. All cases evolved the same microgroves with “valleys and mountains “ as signs of the photodisruption process. Femtosecond laser capsulotomy should be performed before lens fragmentation minimizing the rate of cutting errors. Especially in eyes with advanced cataract, as intracapsular pressure may increase due to lens fragmentation without anterior capsular opening.

To perform capsulorhexis is one of the most important steps in cataract surgery. Since 2010 a femtosecond laser is possible to automize this process and to perform standardized capsulotomies in different sizes of the anterior human lens capsule without a surgical opening of the eye bulb. Main advantage of the laser is the high reproducibility and circularity of this procedure. Even more, it is possible to use femtosecond laser performed capsulotomies to fixate new intraocular lens designs with an exact lens position^1–3^.

Studies have already demonstrated that capsulotomies performed with a femtosecond laser have a repeatable precise size and centration^1–3^. Of note, the use of less energy and larger spot separation can lead to smaller collateral damaged tissue areas along the cutting edges^4^. In previous published SEM analyses, we have already seen that femtosecond laser capuslotomies peformed before lens fragmentation lead to more bridges and tags compared to manual performed procedures. This fact can be a risk factor for radial tears^5,6^. However, Bala et al. found differences in the smoothness in the capsular edge when comparing different laser platforms^7^.

The purpose of our experimental study was to study microanatomical structures of anterior human lens capsule specimens after low energy femtosecond-laser capsulotomy before and after lens fragmentation.

## Methods

In twenty eyes of ten patients with age related cataract, based on LOCS III grading, femtosecond laser-assisted surgery (FLACS) was performed using the Ziemer Z8 platform. Seven patients were female and the average age of all patients was 71 years from 59 to 81. No further ocular comoribities were present. Further inclusion criteria were a minimum pupil size after drug-induced pupil dilation of 7 mm to perform all femtosecond laser-assisted surgical steps.

Femtosecond laser settings were modified in using capsulotomy first procedure (group 1) or eyes receiving capsulotomy after lens fragmentation using a standard six segment profile including two fragmentation rings (group 2). The first eye of each patient was assigned randomely to a group. Randomization was performed with an online randomizer (random.org). The second eye was treated with the different sequence of procedures. One patient (two eyes) of each group showed pseudoexfoliation on the anterior capsule during slitlamp examination. After anterior capsule removal, tissue was immediately fixed in 4.5% formalin and cutting-edge profiles were analysed using scanning electron microscopy (SEM). Only completely extracted and mounted specimens were used for the study.

The study protocol was reviewed and approved by the Institutional Review Board of the department of ophthalmology at Ludwig-Maximilians-University Munich (Ethikkommission LMU). The tenets of the Declaration of Helsinki were followed throughout the study. Informed patient consent was obtained from all study participants.

### Surgical procedure

All femtosecond laser procedures were performed after topical mydriasis and under topical anaesthesia with Conjucain (oxybuprocainhydrchloride) EDO eye drops (Dr. Mann Pharma GmbH, Berlin, Germany) by the same surgeon (WJM) and were all uneventful.

A low energy laser system with high frequency was used for all procedures (Ziemer Z8, Ziemer company, Switzerland). Standard laser pulse energy settings and spot size separation was used for both groups according to the manufacture’s adjustments for femtosecond laser-assisted cataract surgery. Of note, only capsulotomy and fragmentation procedures were performed, whereby the corneal incisions were performed manually in order to exclude further laser related factors that can influence our experimental investigations. For the capsulotomy and lens fragmentation procedure the following laser parameters were used as recommended by the manufacture: Capsulotomy size 5.1 mm, capsulotomy power 110%, Velocity 50 mm/s, Resection height 0.4 mm, lens fragmentation diameter 5.5 mm, lens power 110%, 6 segments, velocity 10.0 mm/s.

The depth and coordinates of the femtosecond laser performed capsulotomies and fragmentation were determined with the live optical coherence tomography (OCT) integrated into the laser system.

### Scanning electron microscopy (SEM)

For scanning electron microscopy (SEM), specimens were fixed in a cacodylate‐buffered solution containing 4% paraformaldehyde (PFA) and 4% glutaraldehyde for 24 h. Subsequent to washing with cacodylate buffer and dehydration in ascending ethanol and acetone series, the samples were critical point dried and sputter coated with gold‐palladium. Images of the complete capsulotomy were captured with an Auriga scanning electron microscope (Carl Zeiss AG), with further focus on three random areas for all specimens.

Primary and secondary endpoints of the study were cell structure, tears in the capsule edge, configuration of the nuclei and cell structure and abnormalities of the capsulotomies.

Thickness profile of specimen images along the capsulotomy was calculated and reproduced with five measurements using ImageJ software (NIH, open source software, USA).

The overall irregularity of the cutting edge was then graded on a scale of 0 to 3 according to the work of Mastropasqua et al.^8^ In brief, a nearly regular cutting edge with only slight irregularities was graded 0, whereas grade 1 indicated a slightly irregular surface with minimal microgrooves, pitting, or notches, grade 2 an irregular surface with minimal microgrooves, pitting, or notches and grade 3 indicated a high irregular surface with microgrooves, pitting, or notches. The irregularity grading was judged independently by two authors (W. J. M. and M. S.). In case of discrepancy the average of both gradings were taken.

### Statistical analysis

The Shapiro–Wilk test was used to test for normal distribution of data. In addition, Mann–Whitney tests were used to statistically compare the differences among the anterior cutting edges of capsulotomy specimens characteristics. Statistical analysis was performed with SPSS software (version 24.0, SPSS Inc., Chicago, IL). For all tests, a p value of < 0.05 was considered statistically significant.

## Results

All femtosecond laser-assisted lens surgeries were uneventful. There was no statistical difference in overall laser treatment time between groups (109 s + /- 34 s, group 1 vs. 116 s ±  29 s, p = 0.32).

In group 1 all performed capsulotomies were “free-floating” with no attachments to the lens capsule, whereas in group 2 two capsulotomies were not “free-floating”. LOCS III grading showed also no difference in cataract manifestation between groups (grade 2–3, *P* = 0.39). All cataracts were nuclear with only a small amount of cortical opacification.

### Scanning electron microscopy analysis

**Figure 1:**
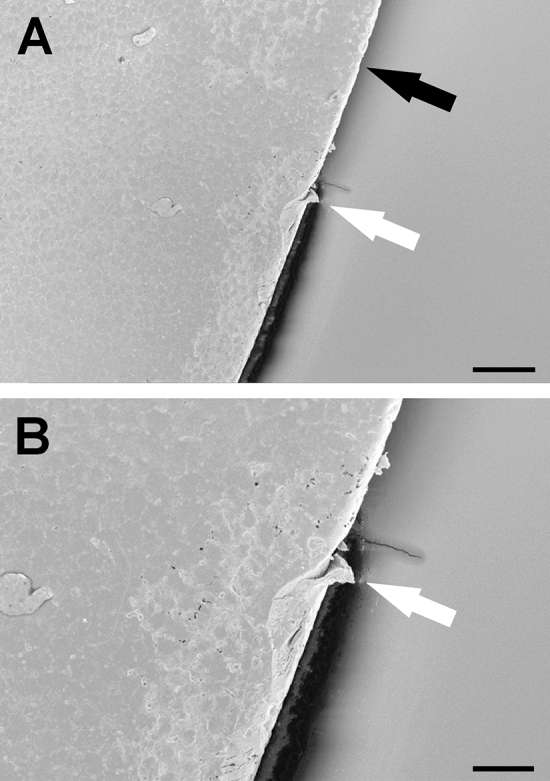
(**A**) and (**B**): SEM sample of a capsulotomy before fragmentation procedure sequence. Smooth cutting edge (black arrow) with only few bridges and grooves (white arrow). Magnification bar for A: 50 µm and B: 25 µm.

**Figure 2:**
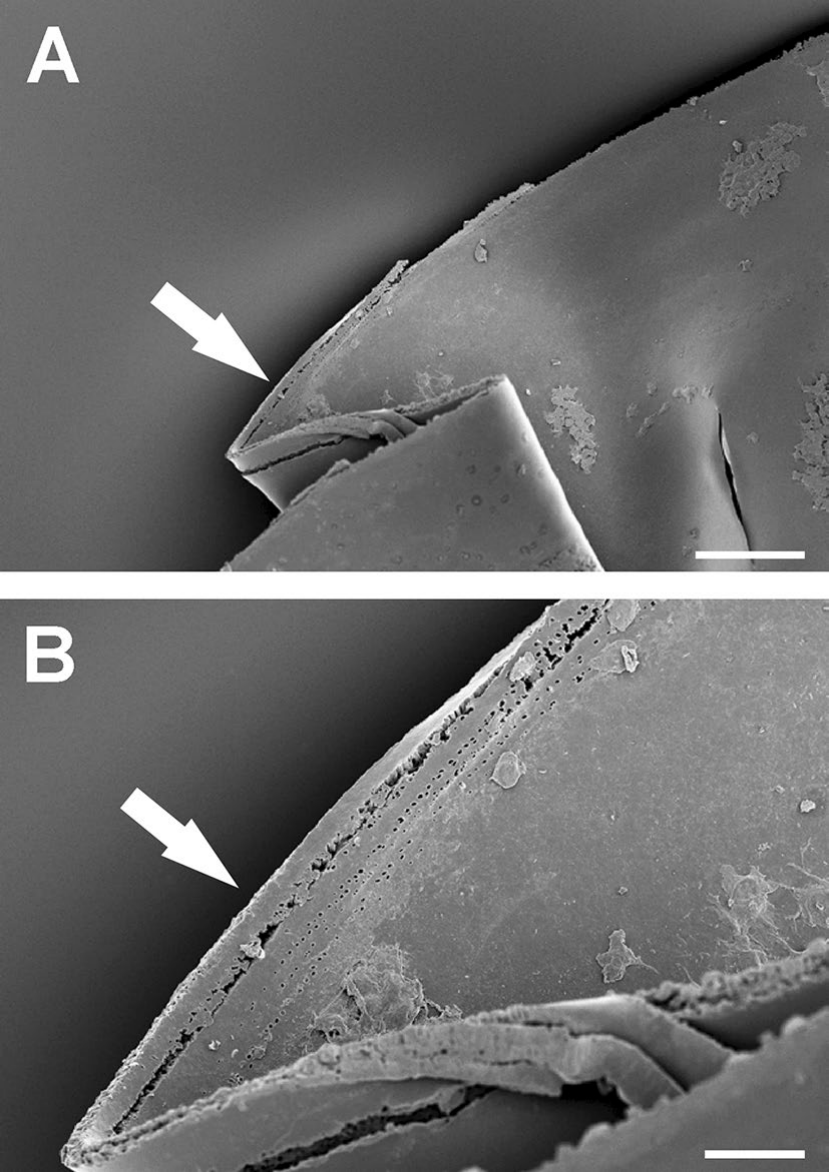
(**A**) and (**B**): SEM sample of a fragmentation before capsulotomy procedure sequence showing a high amount of tissue cutting grooves and bridges (white arrow). The offset laser spot cutting pattern is conspicuous. Magnification bar for A: 100 µm and B: 25 µm.

SEM analysis showed more smooth edges in the first group (Fig. 1A) with a reproducible demarcation line along the cutting edge (Fig. 1B). The second group showed more tags and bridges and a significant number of staggered cutting patterns (7 out of 10 cases, Fig. 2A) Typical findings in group 2 was a multiple contours laser spot pattern due to the circular movement of the laser sequence from posterior to anterior. All of this cases evolved the same microgroves with „valleys and mountains “ as signs of the staggered photodisruption process (Fig. 2B).

The thickness profile was measured with a mean of 4.2 ± 0.51 microns for group 1 and with a mean of 4.60 ± 0.63 microns of group 2, respectively (p = 0.26).

In contrast, the cut surface irregularity showed a significant difference in grading with a value of 1.2 ± 0.89 (range 0–2) for group 1 and 2.1 ± 0.77 (range 0–3) for group 2 (p = 0.037).

### Pseudoexfoliation

**Figure 3:**
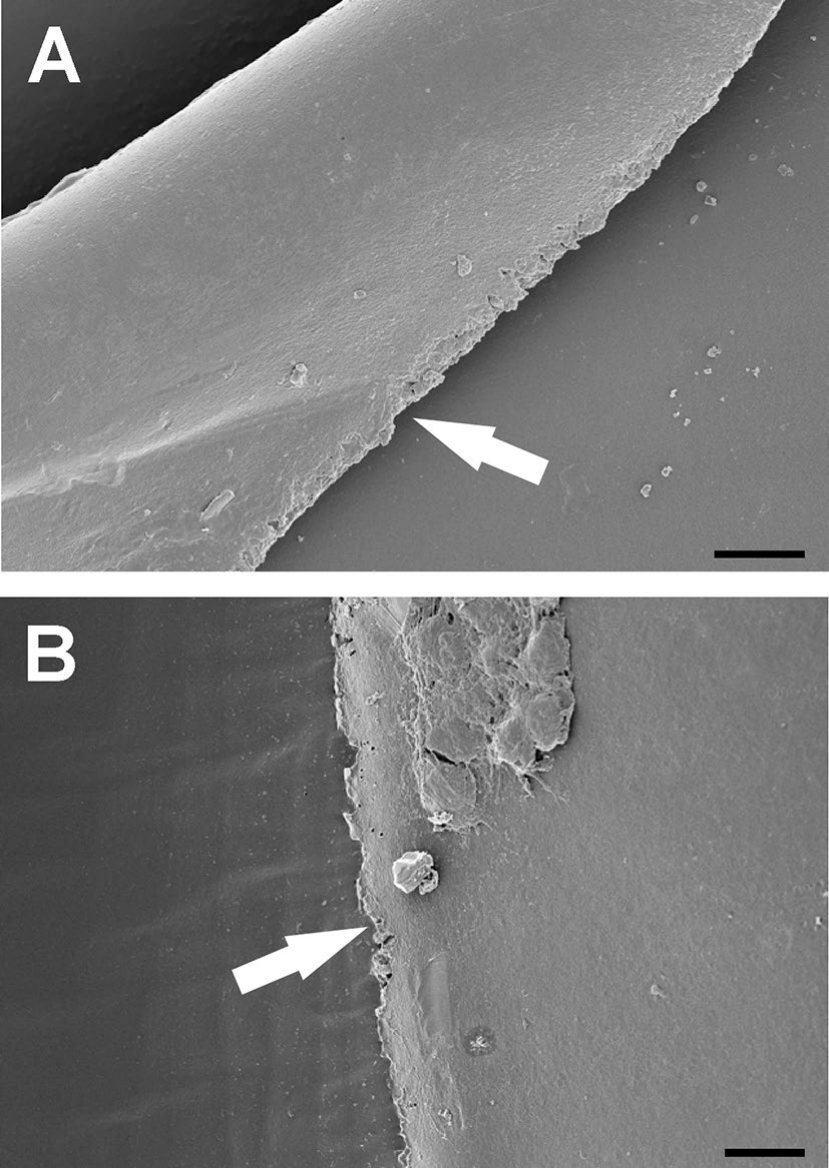
(**A**) and (**B**): SEM samples of a capsulotomy before fragmentation (3a) and vice versa (3b) procedure sequence in pseudoexfoliation cases showing more grooves and bridges in all samples and more sawtooth characteristics in group 2 (3b) (white arrow). Magnification bar for A and B: 10 µm.

Samples with pseudoexfoliation showed more grooves and bridges in all SEM analysis regardless of the chosen sequence of procedures (Fig. 3A,B). PEX samples of group 2 demonstrated in addition a sawtooth pattern as a risk factor for radial tears (Fig. 3B).

## Discussion

Femtosecond laser-assisted lens surgery is a surgical option when performing lens exchange surgery^9^. Different laser platforms are available providing image-guided planning algorithms for lens surgery including corneal incisions, capsulotomy and lens fragmentation. Common to all is the same sequence of these procedures.

A circular, reproducible capsulotomy with planned diameter is crucial for intraocular lens implantation, especially for multifocal lenses^10^. Recent studies reported of a better centration and refractive outcome using femtosecond laser-assisted capsulotomy^10,11^. Moreover, laser guided anterior capsulotomies provide an excellent structure for rhexis-fixated intraocular lenses like the FEMTIS intraocular lens (Teleon, Germany)^12^.

One laser platform, i.e. the Ziemer Z8 femtosecond laser system allows an easy change of the procedure order. We believe that treating lens fragmentation before capsulotomy has a worse outcome on the incision and edges of the anterior lens capsule than when the sequence is reversed.

Some studies could already demonstrate analysis of femtosecond laser-assisted capsulotomy versus manual capsulorhexis^4,13–16^. Tognetto et al. found a similar irregularity level with the Catalys Laser system (1.4 ± 0.63) as we did for Group 1 with the Ziemer Z8 system when performing with the capsulorhexis. The results differ however when compared to the second group in which the sequence of procedures was altered. Interestingly, the irregularity level increased significantly when the fragmentation was performed first. Our hypothesis is, that gas bubbles evolve and different force occur during fragmentation which cause an uneven stress distribution on the capsule which then might cause the higher degree of irregularity. Furthermore, changes in tissue opacity and gas bubble formation in a closed system by performing first the fragmentation might also hinder the laser and cause more irregularity.

A major influence is the laser power and frequency used. Using an improved interface with adapted laser energy, we could already show improved results of incision guidance in capsulotomy with the LenSx laser platform of the company Alcon^17^.

When enlarging the numerical aperture of the focusing optics, the pulse energy threshold for optical breakdown decreases, and cutting with practically no side effects is enabled when using low energy with a high frequency setting^18^.

The present experimental study shows that in the presence of a cataract of intermediate hardness, capsulotomy treatment prior to nucleus fragmentation provides better results in terms of cutting accuracy and reproducibility at the electron microscopic level, especially even in the presence of altered capsular leaflet situations such as pseudoexfoliation.

Limitations of this study are the small sample size and the usage of standard femtosecond laser energy profiles as recommended by the manufacture.

Further studies are necessary to optimize laser energy profiles and OCT imaging to customize laser-based capsulotomy procedure for different stages of cataract formation.

## What was known

- Femtosecond lasers produce precise capsulotomies.
- Laser-based capsulotomy has more tissue bridging and hairline fractures compared to manual capsulotomy.

## What this paper adds

- A low energy laser platform with high frequency produces a smooth cutting edge profile.
- Cutting profile of capsulotomy samples with capsulotomy before fragmentation procedure sequence showed less tissue bridges and grooves and no laser spot deviation in the cutting pattern as in a vice versa procedure sequence.
- Femtosecond laser-assisted capsulotomy should be performed before lens fragmentation in term of cutting safety profile.

## Data Availability

The datasets generated and analysed during the current study are available from the corresponding author on reasonable request.
